# NLRX1 Deficiency Alters the Gut Microbiome and Is Further Exacerbated by Adherence to a Gluten-Free Diet

**DOI:** 10.3389/fimmu.2022.882521

**Published:** 2022-04-28

**Authors:** Holly A. Morrison, Yang Liu, Kristin Eden, Margaret A. Nagai-Singer, Paul A. Wade, Irving C. Allen

**Affiliations:** ^1^ Department of Biomedical Sciences and Pathobiology, Virginia-Maryland College of Veterinary Medicine, Virginia Tech, Blacksburg, VA, United States; ^2^ Eukaryotic Transcriptional Regulation Group, Epigenetics and Stem Cell Biology Laboratory, National Institute of Environmental Health Sciences, Research Triangle Park, Durham, NC, United States; ^3^ Department of Basic Science Education, Virginia Tech Carilion School of Medicine, Roanoke, VA, United States

**Keywords:** pattern recognition receptors (PRRs), NOD-like receptors (NLRs), gluten free diet, microbiome, dysbiosis, metabolites, probiotics

## Abstract

Patients with gluten sensitivities present with dysbiosis of the gut microbiome that is further exacerbated by a strict adherence to a gluten-free diet (GFD). A subtype of patients genetically susceptible to gluten sensitivities are Celiac Disease (CeD) patients, who are carriers of the HLA DR3/DQ2 or HLA DR4/DQ8 haplotypes. Although 85-95% of all CeD patients carry HLA DQ2, up to 25-50% of the world population carry this haplotype with only a minority developing CeD. This suggests that CeD and other gluten sensitivities are mediated by factors beyond genetics. The contribution of innate immune system signaling has been generally understudied in the context of gluten sensitivities. Thus, here we examined the role of NOD-like receptors (NLRs), a subtype of pattern recognition receptors, in maintaining the composition of the gut microbiome in animals maintained on a GFD. Human transcriptomics data revealed significant increases in the gene expression of multiple NLR family members, across functional groups, in patients with active CeD compared to control specimens. However, *NLRX1* was uniquely down-regulated during active disease. NLRX1 is a negative regulatory NLR that functions to suppress inflammatory signaling and has been postulate to prevent inflammation-induced dysbiosis. Using *Nlrx1^-/-^* mice maintained on either a normal or gluten-free diet, we show that loss of NLRX1 alters the microbiome composition, and a distinctive shift further ensues following adherence to a GFD, including a reciprocal loss of beneficial microbes and increase in opportunistic bacterial populations. Finally, we evaluated the functional impact of an altered gut microbiome by assessing short- and medium-chain fatty acid production. These studies revealed significant differences in a selection of metabolic markers that when paired with 16S rRNA sequencing data could reflect an overall imbalance and loss of immune system homeostasis in the gastrointestinal system.

## Introduction

Adherence to a gluten-free diet (GFD) is currently the only treatment available to patients with gluten sensitivities like Celiac Disease (CeD) and non-celiac gluten sensitivity (NCGS). Although being an extensive dietary change, gluten exclusion has yet to be meaningfully evaluated in host health. Also of significant interest, current “fad diets” promote the dietary exclusion of gluten in healthy individuals without any prior diagnoses of gastrointestinal disorders with the generally unsubstantiated claims that this lifestyle change promotes gut health and anti-inflammatory properties. However, wheat-derived oligosaccharides are likely prebiotic in nature (1). Therefore, entire elimination of gluten-containing foods, namely wheat-based sources, is anticipated to have dysbiotic effects by impairing bacterial metabolic activity and subsequently metabolite production, such that gut barrier function and overall host health are compromised.

Upon exposure, typically from dietary intake, gluten-sourced gliadin and glutenins are incompletely digested and remain active in the gastrointestinal tract in patients with gluten sensitivities (2–6).These partially degraded gluten fragments and peptides transverse the epithelial barrier to elicit a strong adaptive T-cell immune response (7–9). Translocating to the lamina propria of the small intestine, type 2 transglutaminase (TG2), a CeD autoantigen, deamidates partially-digested gluten peptides, causing TG2 to autocatalyze itself and increasing the affinity of gluten peptides to DQ2/8 to antigen presenting cells, thus inducing T-cell driven auto-destruction of small intestinal epithelia (10). This induces the production of pro-inflammatory cytokines including IFN-γ, TNF, IL-15, and IL-8 and decreases anti-inflammatory cytokines like IL-10 (11, 12). Adaptive immune system dysfunction has been widely studied in CeD. However, it is also clear that elements of the innate immune system also significantly contribute to disease pathogenesis. For example, particular gliadin peptides have enhanced resistance to degradation, particularly gliadin P31-43 (13). These peptides robustly activate innate immune responses *via* activation of IFN-α and TLR7/MyD88 signaling. Mechanistically, gliadin fragments mimic a viral response by activating TLR7 signaling impairing endocytic trafficking and subsequently activating NF-κB signaling (13). This indicates a major contribution of dysregulated innate immune responses in gluten sensitivities and likely involvement of nucleotide-binding domain and leucine-rich repeat-containing proteins (NLRs) which negatively regulate NF-κB signaling.

Tightly regulated NLR signaling is critical for maintaining the host defense against infection and dysbiosis, while activating the host immune response against damage that may accrue as a result of GFD-induced dysbiosis. This form of pattern recognition receptor (PRR) signaling functions to maintain symbiosis of the gut microbiome by surveilling the gut mucosa for potentially breached mucosal barriers and infiltration of pathogenic, opportunistic bacteria by recognizing highly conserved pathogen-associated molecular patterns (PAMPs) and damage-associated molecular patterns (DAMPs). NLRs can be divided into multiple subgroups, including inflammasome forming NLRs (14–19) and regulatory NLRs (20–25). NLRX1 and NLRP12 are negative regulators of inflammation that impact a variety of inflammatory pathways and have been implicated in several diseases (26–40) and may be further implicated in patients with gluten sensitivities like CeD and NCGS. As CeD patients present with dysbiosis that is further exacerbated by adhering to GFD (41, 42), tightly regulated NLR signaling is likely critical for ensuring symbiosis of the gut microbiota both prior to and following adherence to GFD and protecting against gastrointestinal pathologies, specifically related to gluten sensitivity.

NF-κB signaling is attenuated by NLRX1 and NLRP12 and this signaling pathway has been previously demonstrated as being dysregulated in CeD patients. NLRX1 is a negative regulator of the canonical NF-κB pathway by associating with TRAF6 and IKK (22, 31, 36, 43). NLRP12 functions to negatively regulate noncanonical NF-κB signaling by associating with TRAF3 and NIK (28). These NLRs directly attenuate inflammatory signaling in the gut and have been found to be dysregulated in inflammatory bowel disease and colitis-associated cancer (22, 28, 44, 45). Their role has been evaluated in maintaining the host immune response to pathogenic microbes and maintaining the colonic microbiota. NLRX1 has been previously demonstrated as a key regulator in maintaining host-gut microbiota interactions and protecting against gut dysbiosis in colitis (37). Loss of NLRX1 in mice results in impaired gene expression related to tight junction of the intestinal epithelial barrier, which further implicates the expansion of colitogenic bacteria including *Veillonella* and *Clostridiales* upon increased permeability (37). In an oral bacterial infection model, NLRX1 activates the NLRP3 inflammasome following *Fusobacterium nucleatum* infection to elicit a host immune response for bacterial clearance (46). The exact mechanism for microbial responses following NLRX1 activation has been studied extensively in viral work where NLRX1 attenuates Type 1 Interferon production and induces autophagy following viral infection (47). Deletion of NLRP12 in mouse models is associated with dysbiosis of the gut characterized by decreased commensal bacteria including *Lachnospiraceae* and increased colonization of enteropathogenic bacteria namely *Erysipelotrichaceae* (28, 34, 38, 48). Current studies have yet to identify the exact molecular underpinnings by which NLRP12 regulates the gut microflora with studies highlighting both the hematopoietic and non-hematopoietic compartments (28, 49). Interestingly, NLRP12’s role as a negative regulator in dampening the host immune response has been demonstrated to perpetuate bacterial infection, where decreased production of proinflammatory cytokines and antimicrobial molecules prevents clearance of *Salmonella* in a mouse infection model (50). Due to varying insights associated with prior publications, more work is necessary to better define the role of these regulatory NLRs in maintaining gastrointestinal health and disease.

Currently, there is speculation as to whether dysbiosis is a cause or consequence of these etiologies and whether these microbial signatures are genetically inherent or determined by environmental factors like diet. Genetically inherent factors may include depletion of regulatory NLR signaling, such as deficits of NLRX1 or NLRP12, while the primary environmental factor of interest is adherence to GFD. While the gut microbiome of NLRX1- and NLRP12-deficient mice have been evaluated in the context of colitis (28, 32, 34, 37, 49), their potential predisposition towards GFD has yet to be meaningfully demonstrated. Further, innate immune signaling has been overlooked in the context of CeD and NCGS pathology. We therefore hypothesize that the regulatory NLRs, NLRX1 and NLRP12, have a pertinent role in maintaining proper innate immune responses and microbiome homeostasis in the context of GFD, which is of relevance to CeD and NCGS. To evaluate this hypothesis, we assessed the gut microbiome composition and metabolic profile of *Nlrx1^-/-^* and *Nlrp12^-/-^* mice maintained on a GFD compared to normal diet to characterize the influence of NLR-deficiency in maintaining the gut microbiome. Our findings imply that loss of NLR signaling can be significantly exacerbated by simple dietary changes, such as switching to GFD, and may contribute to inflammatory diseases like gluten sensitivities.

## Materials and Methods

### Experimental Animals

The generation and characterization of *Nlrx1^-/-^ and Nlrp12^-/-^* mice have been previously described (22, 26). Following IACUC approval by Virginia Tech, all experiments were conducted in accordance with the NIH Guide for the Care and Use of Laboratory Animals. Animal housing conditions include the room temperature being maintained at 22°C with humidity ranging between 30-70%. The light cycle in the room is 14 hours light to 10 hours dark. Wild-type, *Nlrx1^-/-^, and Nlrp12^-/-^* mice were housed in standard specific pathogen-free (SPF) conditions in animal vivariums located at the Virginia-Maryland College of Veterinary Medicine, which is American Association for Laboratory Animal Care (AALAC) accredited. SPF status of the mouse colony is routinely checked using standard best practices. All experiments were conducted with male and female 6-month old mice that derived from a C57Bl/6J background. GFD wild-type, *Nlrx1^-/-^*, and *Nlrp12^-/-^* mice were bred and fed commercially-available rodent gluten-free diet (*Research Diets #AIN-76A)* for at least three generations. Control mice were maintained on normal diet (*Tekland Global 18% Protein Rodent Diet)* throughout generations (**Supplemental Table 1**).

### Fecal Samples

Prior to harvest, necropsy tools were washed and sterilized *via* autoclave with a designated set of tools used for each genotype. Mice were euthanized in a biological safety cabinet at necropsy. Colonic contents were collected and immediately stored on dry ice. Long-term storage of samples were kept at -80°C.

### 16S rRNA Gene Sequencing and Data Analysis

One fecal pellet from each individual mouse was prepared for DNA extraction and 16S rRNA gene sequencing (n = 6-7 animals per group). Genomic DNA was extracted from fecal pellets using the DNeasy PowerSoil HTP 96 Kit according to the manufacturer’s protocol. Genomic DNA was suspended in 100 µl of manufacturer supplied C6 solution. Extracted DNA was stored at -20°C until further processing. PCR amplification of a region of the mitochondrial 16S rDNA gene was performed on the extracted DNA sample using forward and reverse primers. The primers used include the forward primer GTGYCAGCMGCCGCGGTAA and reverse primer GGACTACNVGGGTWTCTAAT. The forward and reverse primers both contained a 5’ adaptor sequence for Illumina sequencing. The 25 µl total volume PCR master mix was prepared for each sample using Promega PCR Master Mix Specifications: 12.5 µl Master Mix, 0.5 µl of each primer, 1.0 µl gDNA, and 10.5 µl DNase/RNase-free H2O. DNA was amplified under the following cycle conditions: initial denaturation at 95°C for 5 minutes, followed by 35 cycles of 45 seconds at 95°C, 1 minute at 50°C, and 90 seconds at 72°C, and a final elongation at 72°C for 10 minutes. Sample library pools were subsequently sequenced on an Illumina MiSeq (San Diego, CA) using a v2 500-cycle kit. Proper quality control was conducted prior to sequencing. Sequencing data was analyzed using Mothur V.1.40.4 (51) to group 16S rRNA sequences into OTUs using 97% similarity. Classifications were determined to a group by comparing sequences to the Silva database 132. Relative abundances of bacterial phyla and family were determined per sample through classified OTUs and all taxonomic plots were generated using R (version 4.0.5) Samples were normalized to the same number to adjust for variation in sequencing depth. Principal coordinate analysis was used to assess community similarity among all samples. Weighted unifrac distances between communities were displayed in a two-dimensional space (52). Linear discriminant analysis effect size (LEfSe, Galaxy version 1.0; https://huttenhower.sph.har-vard.edu/galaxy/) was performed to compare the differential bacterial abundance using default settings (53). This analysis was performed with the default setting (Alpha value for the factorial Kruskal-Wallis test: 0.05; Threshold on LDA score: 2).

### Short- and Medium-Chain Fatty Acids Identification and Quantification

Fecal pellets were harvested following the above protocol. Approximately 100mg fecal samples were weighed in a biological safety cabinet.1 mL sterile molecular-grade water (Sigma-Aldrich) was added to each sample. At room temperature, fecal samples were placed on a tilt table for 4 hours and vortexed once every hour to disintegrate larger fecal pieces. Following final vortex, samples were allowed to settle for approximately 30 minutes and 900 μL of supernatant was collected for analysis. 10 μL of 85% phosphoric acid was added to the sample to achieve 1% acid concentration. Samples for VFA analysis were passed through a 0.45 μm nitrocellulose membrane filter and frozen prior to analysis to avoid capillary blockage. Upon analysis, samples were defrosted and centrifuged prior to detection. Volatile fatty acid (VFA) analysis was performed using an Agilent 6890 gas chromatograph (Agilent, Wilmington, DE, USA) equipped with a split injector and a flame-ionization detector. Chemstation software was utilized to perform data analysis. NukolTM fused silica 15 m × 0.53 mm capillary column with 0.5-μm film thickness was used to perform VFA separation. The carrier gas helium flow rate was 15 mL/min with a split ratio of 2:1. The oven temperature was held constant at 80°C for 3 min, increased to 140°C at a rate of 6°C per minute, where it was held constant for 1 min. Temperatures were maintained at 200°C for the injector and 250°C for the detector. VFA standards were used to generate a standard curve. VFAs are reported as mg/L. Normalization of readings were performed by dividing by weight of total feces collected in milligrams.

### Human Transcriptomic Data Analysis

Expression of genes related to NLR signaling in CeD patients was evaluated using publicly accessible gene expression datasets, as previously described (54–59). Data shown were generated from RNA-sequencing data from duodenal biopsies obtained from 51 CeD patients and 45 healthy controls, as previously published (60). Individual gene expression was based on transformed data, converted to relative gene expression for each individual target (54–59). Pathway analysis was conducted using Ingenuity Pathway Analysis (IPA) and CompBio (54–59). Data were analyzed using causal analysis approaches in IPA (61). Gene expression and pathway analysis in human subjects were conducted using the following array data series (available through the National Center for Biotechnology Information: https://www.ncbi.nlm.nih.gov/): GSE134900.

### Statistical Analysis

Data analysis was performed using GraphPad Prism version 9.2.0 (Graph- Pad, San Diego, CA, USA) to assess statistical significance and calculate correlation. Student two-tailed t-test was calculated for comparisons between two experimental groups. One-way and two-way ANOVA were calculated for multiple comparisons, with Mann-Whitney or Tukey post-tests for multiple pairwise examinations as appropriate. Changes were identified as statistically significant with p <0.05. Mean values were reported with standard error of the mean. Statistical analyses for α-diversity and β-diversity were compared by nonparametric Mann-Whitney U tests and nonparametric multidimensional ANOVA. Distance-based redundancy analysis determined the contribution of different variables to microbiota profile variations.

## Results

### Human Transcriptome Analyses Indicate Significant Dysregulation of NLR Signaling in CeD Patients

**Figure 1:**
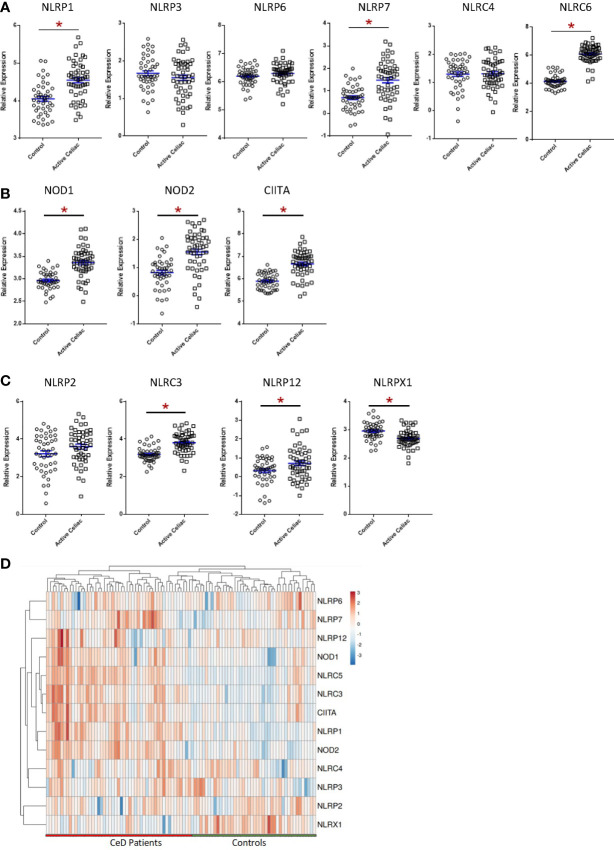
Significant Dysregulation of NLR Signaling in Active CeD Patients. **(A)** Inflammasome-forming NLR signaling trended higher in active CeD patients than controls, with significant variation observed in NLRP1, NLRP7, and NLRC5. **(B)** Gene expression of the regulatory NLRs NOD1, NOD2, and CIITA were significantly upregulated in active CeD patients. **(C)** Gene expression of the negative regulatory NLRs also trended higher in active CeD patients, with significant upregulation observed in NLRC3 and NLRP12. NLRX1 is the only negative regulatory NLR that was significantly downregulated. **(D)** Gene expression heatmap of assorted NLRs in CeD patients. *p < 0.05.

To evaluate NLR signaling in CeD patients, we conducted both biased and unbiased evaluations of publicly available transcriptomic datasets from human duodenal biopsy samples (GSE134900). This dataset included 51 patients with active CeD and 45 control patients, as previously defined (60). Thirteen NLR family members were represented on the human arrays (**Figure 1**). While this is not a comprehensive list of the full NLR family, representatives from each of the inflammasome and regulatory subsets of NLRs are present on the array, as well as the majority of adaptors, caspases, and modulators of each NLR signaling pathway. In the human CeD patients with active disease, we observed a significant increase in NLR inflammasome signaling (**Figure 1A**; **Supplemental Figure 1B**). Specifically, genes associated with the NLRP1, NLRP7, and NLRC5 inflammasomes were significantly up-regulated in the CeD patients (**Figure 1A**; **Supplemental Figure 1B**). Likewise, we also observed significant up-regulation of the regulatory NLRs associated with pro-inflammatory signaling, including NOD1, NOD2, and CIITA (NLRA) signaling in patients with active CeD (**Figure 1B**; **Supplemental Figure 1**).

**Figure 2:**
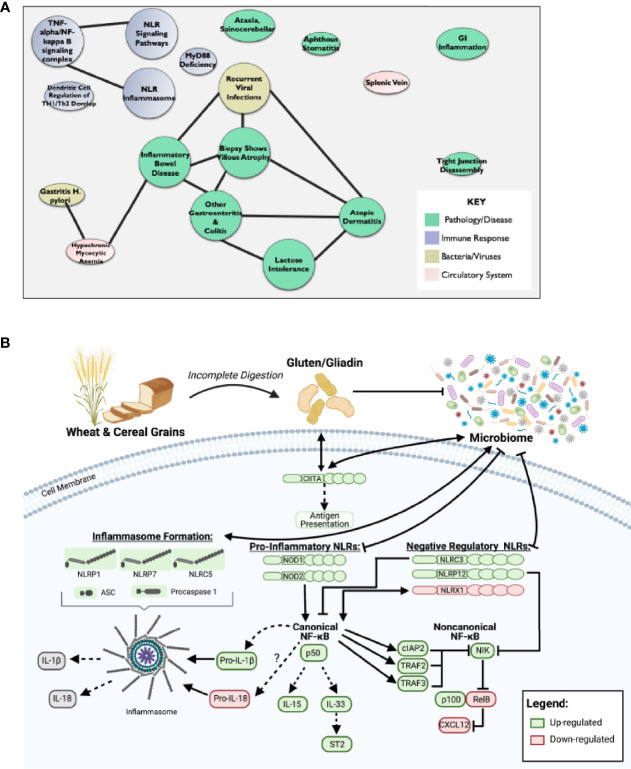
Dysregulation of NF-κB Pathway Downstream of Impaired NLR Signaling May Potentiate CeD Pathogenesis. **(A)** CompBio AI algorithm predicted increased NF-κB/TNF signaling, NLR inflammasome signaling, and regulatory NLR signaling that is consistent with CeD pathology. **(B)** Schematic representative of IPA data output predicts that partial digestion of gluten is recognized as a DAMP by PRRs to signal NLRs in CeD. This results in downstream activation of canonical NF-κB and inflammasome formation, while inhibiting noncanonical NF-κB.

In addition to the pro-inflammatory NLRs, we also observed differences in signaling associated with the regulatory NLRs that attenuate inflammation. We did not see any significant changes in *NLRP2* (**Figure 1C**). However, we did observe significant increases in *NLRC3* and *NLRP12* and significantly downregulated *NLRX1* expression (**Figure 1C**). NLRX1 was the only NLR represented on the array that was significantly downregulated and demonstrated a unique expression pattern in the CeD patients as illustrated in the heatmap (**Figure 1D**). To better define the mechanisms and pathways impacted by the changes in gene expression, selected targets were analyzed using the CompBio pathway analysis tool, as previously described (58). This program utilizes an artificial intelligence algorithm to identify biological functions most likely impacted by complex changes in gene expression in large datasets. Consistent with the observations discussed above, CompBio predicted increased NF-κB/TNF signaling, NLR inflammasome signaling, and regulatory NLR signaling pathways (**Figure 2A**). Consistent with CeD, we also observed a significant change in dendritic cell regulation of TH1/TH2 development, GI Inflammation, and epithelial cell tight junction disassembly (**Figure 2A**). Villous atrophy formed a central node in the largest cluster, that also included atopic dermatitis, lactose intolerance, colitis, and IBD (**Figure 2A**). Likely due to the up-regulation of TLR7 and several NLRs associated with host-pathogen interactions, specifically virus recognition, the pathway analysis revealed the general up-regulation of signaling pathways consistent with pathogen associated molecular patterns (PAMPs) and damage associated molecular patterns (DAMPs), which are reflected in the recurrent viral infection node (**Figure 2A**). These findings were confirmed using IPA. The schematic shown in **Figure 2B** is representative of the gene expression data output generated by IPA, showing the predicted biological functions associated with the global changes in gene expression focused on the NLR signaling data (**Figure 1**; **Supplemental Figure 1**). Together, these data suggest that incomplete digestion of gluten/gliadin is potentially recognized as a DAMP by pattern recognition receptors in the context of CeD. Likewise, most of the NLRs identified have significant roles in maintaining microbiome homeostasis, with dysregulation often resulting in dysbiosis (34, 37, 62–64). Thus, the gene expression changes are further predicted to significantly impact microbiome homeostasis in CeD patients, as predicted in the pathway analysis (**Figure 2B**).

### GFD Distinctively Shifts the Gut Microbiota Composition in Mice With Intact NLR Signaling

**Figure 3:**
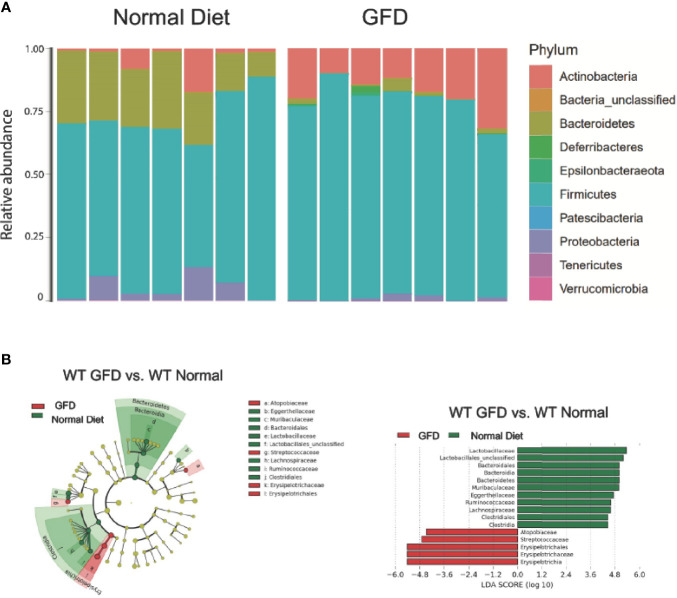
Mice with Intact NLR Signaling Display a Distinctive Microbiome Depending on Diet-type. **(A)** The microbiomes of mice with intact NLR signaling are dominated by the Firmicutes phyla. The second most abundant phyla is driven by diet type with normal diet being characterized by Bacteroidetes and GFD by Actinobacteria. **(B)** LEfSe differential analysis displaying LDA scores reveals that significant differences exist between the microbial populations (GFD, red and normal diet, green. The graph was generated using the LEfSe program. GFD (red), *n* = 7*;* normal diet (green), *n* = 7.

Microbiome changes have been explored in CeD patients and can significantly impact NLR signaling (60). The most effective therapy for CeD is a gluten-free diet (GFD). However, the complete removal of gluten is also likely to impact the microbiome in individuals on a GFD. Current studies have yet to investigate the impact GFD has on otherwise healthy individuals void of gluten sensitivities. In an effort to better define microbiome changes associated with GFD, we compared the microbiomes based on diet in wild-type mice with normal, intact NLR signaling (**Figure 3**) and predicted that this dietary change will have minimal impacts on a robust healthy gut microbiome.

We observed that mice maintained on GFD demonstrate significant shifts in the microbial ecology within the GI niche compared to mice maintained on the normal diet (**Figure 3**). These findings demonstrate the effects a GFD has on otherwise healthy individuals. We demonstrate here that bacteria belonging to the Firmicutes phylum dominates the microbiome, while the second most abundant phylum is correlated with diet. The second most abundant phyla for mice fed a normal diet were Bacteroidetes and Actinobacteria for GFD-fed mice (**Figure 3A**). Cladogram and LEfSe differential analysis displaying bacterial abundance showed that mice fed a normal diet exhibited significant variation between wild-type mice fed GFD (**Figure 3B**), whereby mice on a GFD are increasingly characterized by Atopobiacae, Streptococcaceae, and Erysipelotrichaceae. Investigation at a family level indicates that wild-type mice fed a normal diet had a significantly higher abundance of bacteria belonging to the Lactobacillaceae family and unclassified Lactobacillales which are normally associated with beneficial health benefits and utilized as probiotics (**Figure 3B**).

**Figure 4:**
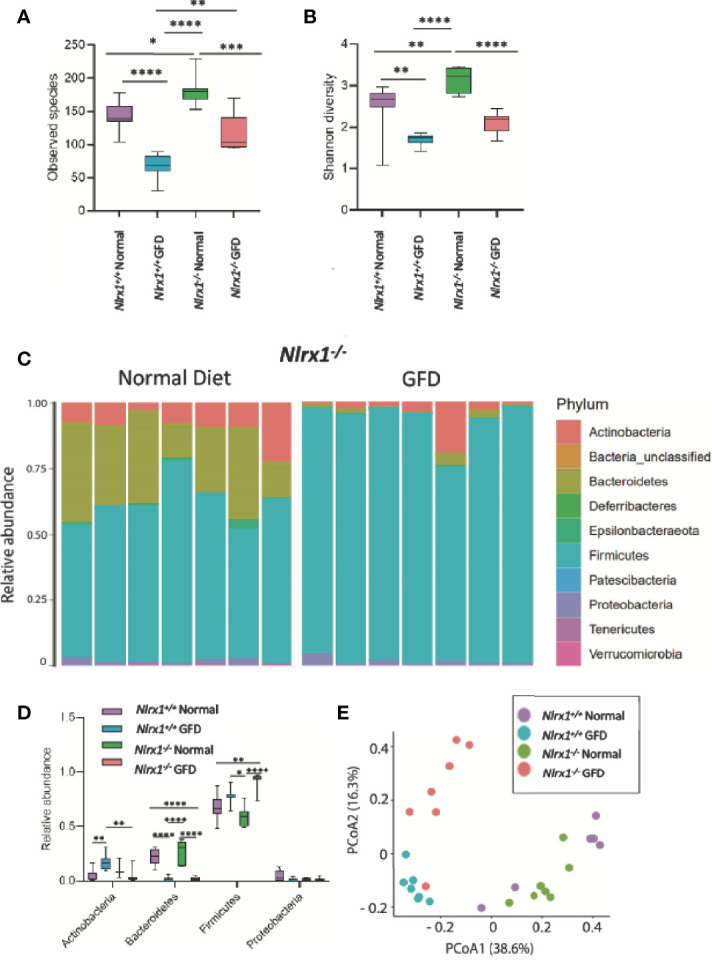
NLRX1-deficient Mice Have Inherently Unique Microflora Diversity. **(A)** *NLRX1^-/-^* mice have significantly increased observed species of bacteria when fed a normal diet compared to GFD. **(B)** Assessment of Shannon Diversity depicts *NLRX1^-/-^* mice to have significantly decreased diversity on a GFD compared to a normal diet. **(C)** The most abundant phyla present in the gut microbiota is Firmicutes. Bacteroidetes is the second most abundant phyla. Relative abundance is driven by diet, as these levels were significantly diminished in GFD mice. **(D)** There is significant variation in relative abundance of phyla present amongst the different treatment groups. **(E)** There is a clear clustering of the groups in PCoA analysis with clusters being apparent for both genetic status and diet type. GFD, *n* = 7*;* normal diet, *n* = 7, *p < 0.05, **p < 0.01, ***p < 0.001, ****p < 0.0001.

Further, mice fed a GFD had significantly decreased overall count of observed bacterial species and Shannon diversity (**Figures 4A, B**), highlighting a robust shift in the microbiota following GFD administration, wild-type mice fed a GFD had nearly 2-fold less observed species than those fed a normal diet (**Figure 4A**). Consistently lacking in diversity, wild-type mice administered GFD had significantly increased relative abundance of Actinobacteria that expanded in the community, while Bacteroidetes was reciprocally decreased (**Figure 3A**, **4D**). Mice fed GFD also had significantly reduced Shannon Diversity, which demonstrates that both the abundance and evenness of species is reduced in this microbial community (**Figure 4B**). Together, these data indicate that a GFD significantly alters the bacterial composition of the gut microbiome in otherwise healthy mice.

### Gut Microbiome of NLRX1-Deficient Mice Has Genetically-Inherent Composition Poised Towards Dysbiosis and Is Exaggerated by GFD

Based on the findings that GFD skews the microbiome towards dysbiosis and that CeD patients have significantly altered NLR signaling predisposing individuals towards gastrointestinal disorders (41, 60), we next sought to evaluate the understudied negative regulatory NLRs in modulating the gut microflora *in vivo* using NLR-deficient mouse models following GFD. Our attention first focused on NLRX1, the only NLR found to be significantly downregulated in human CeD patients (**Figure 1C**). We hypothesized that the altered pattern recognition receptor (PRR) signaling and concurrent hyperinflammatory innate immune system signaling associated with loss of NLRX1 (22, 37) would result in significant alterations in the bacterial composition of the microbiome with populations highly composed of pathogenic/opportunistic bacteria. Based on our findings in **Figure 3**, we further postulated that the GFD would amplify this shift in the microbiome.

To test this hypothesis, we utilized *Nlrx1^+/+^* and *Nlrx1^-/-^* littermates (22), maintained on either normal diet or the GFD as described in the methods. We observed a significant decrease in the number of species in the gut microbiome in the GFD *Nlrx1^-/-^* mice compared to counterparts fed normal diet (**Figure 4A**). We also observed a significant difference between the *Nlrx1^+/+^* GFD mice and *Nlrx1^-/-^* GFD mice, where the *Nlrx1^+/+^* animals on the GFD had the lowest levels of species diversity (**Figure 4A**). A significant difference was noted between *Nlrx1^+/+^* and *Nlrx1^-/-^* mice on the normal diet in number of observed species (**Figure 4A**). The results were further confirmed in the Shannon diversity data; however, no difference was observed between the *Nlrx1^+/+^* GFD mice and *Nlrx1^-/-^* GFD mice (**Figure 4B**). Our data further revealed the relative abundance of bacteria belonging to the phyla Bacteroidetes is significantly decreased and Firmicutes is significantly increased in the *Nlrx1^-/-^* mice on the GFD compared to the *Nlrx1^-/-^* mice on the normal diet (**Figures 4C, D**). Principle Coordinate analysis (PCoA) further confirmed that the microbiomes of the *Nlrx1^+/+^* and *Nlrx1^-/-^* mice are inherently unique and clustered separately upon GFD, with clustering not as strongly apparent for mice on the normal diet (**Figure 4E**; **Supplemental Table 2**). This suggests that diet type and genotype status contribute to microbial community composition in the gut (**Figure 4E**).

**Figure 5:**
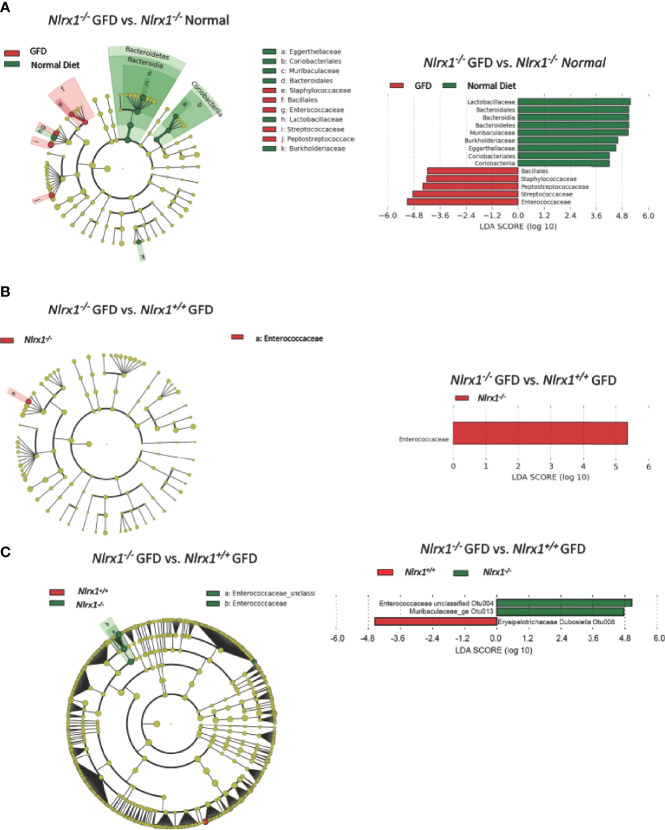
Comparison of the Effects of GFD on the Microbiome of NLRX1-deficient Mice. **(A)** Cladogram displaying differential bacterial abundance in *NLRX1^-/-^* GFD (red) and normal diet (green). LEfSe differential analysis displaying bacterial abundance in in *NLRX1^-/-^* GFD (red) and normal diet reveals significant differences exist between the microbial populations at the family level. The graph was generated using the LEfSe program. *NLRX1^-/-^* GFD (red). **(B)** Cladogram and LEfSe analysis displaying differential bacterial abundance in *NLRX1^-/-^* (green) and mice with intact NLRX1 signaling (red) following GFD at family level **(C)** Cladogram displaying differential bacterial abundance in *NLRX1^-/-^* (green) and mice with intact NLRX1 signaling (red) following GFD at OTU level. LEfSe differential analysis displaying bacterial abundance in in *NLRX1^-/-^* (green) and wild-type mice (red) reveals miniscule differences exist between the microbial populations. The graph was generated using the LEfSe program. n = 7 mice per group.

We next performed LEfSe analysis to determine the differentially abundant taxa comparing *Nlrx1^+/+^* and *Nlrx1^-/-^* mice fed normal and GFD (**Figure 5**). At the phylum level, the microbiome of *Nlrx1^-/-^* mice fed a normal diet are characterized by Lactobacillaceae, Bacteroidales, Bacteroidia, Bacteroidetes, Muribaculaceae, Burkholderiaceae, Eggerthellaceae, Coriobacteriales, and Coriobacteria (**Figure 5A**). The microbiome of *Nlrx1^-/-^* mice on the GFD are characterized by bacteria belonging to the Bacillales, Staphylococcaceae, Peptostreptococcaceae, Streptococcaceae, and Enterococcaceae (**Figure 5A**). Compounding the differences between the GFD and normal diet in the *Nlrx1^-/-^* mice, we also observed that key bacterial populations were found to be differentially abundant between *Nlrx1^-/-^* mice and *Nlrx1^+/+^* mice on the GFD in the LEfSe analysis, indicating the genetically inherent microbiome of NLRX1-deficient mice is not restored by a GFD. We observed a significant increase in the presence of Enterococcaceae in the *Nlrx1^-/-^* mice compared to the *Nlrx1^+/+^* animals (**Figure 5B**). Increases in Enterococcaceae are known risk factors for increased susceptibility to pathogenic bacteria infection, such as *C. difficile* (65) and enhanced gastrointestinal inflammation (66). At the OTU level, the microbiota of GFD *Nlrx1^-/-^* mice had significantly increased levels of the Enterococcaceae unclassified Otu004 and Muribaculaceae_ge Otu013, (**Figure 5C**). The GFD *Nlrx1^+/+^* had increased characterization of Erysipelotrichaceae Dubosiella Otu 008(**Figure 5C**). While most of these bacteria are commensal, the overrepresentation of *Enterococcus* spp. in the *Nlrx1^-/-^* mice fed GFD indicates a unique microbial fingerprint for these NLR-deficient mice.

### Microbiota Composition of NLRP12-Deficient Mice Fed GFD Resembles Community Structure of Mice With Intact NLR Signaling

**Figure 6:**
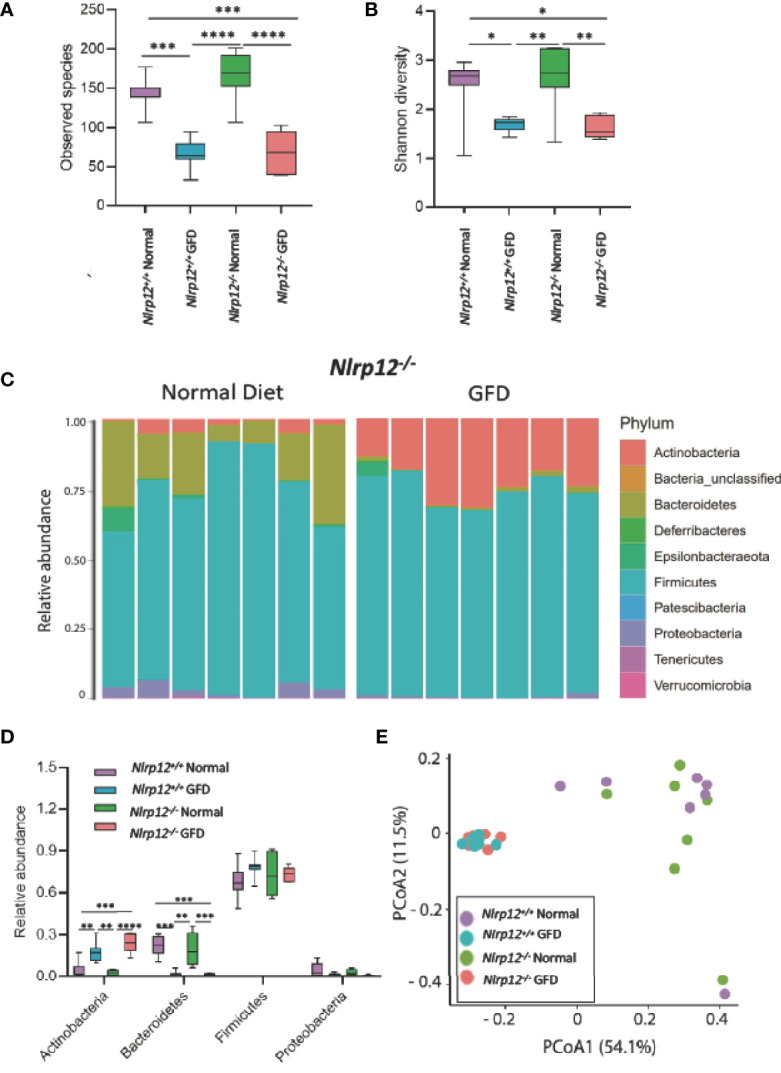
Absence of NLRP12 Has Minimal Effect on Gut Microflora. **(A)** There is no significant variation between *NLRP12^-/-^* mice and wild-type mice in the observed species present, either for normal diet or GFD. **(B)** This trend is also observed in Shannon Diversity. **(C)** Upon assessment of the relative abundance of phyla in the NLRP12^-/-^ gut microbiome, the most abundant phyla present is Firmicutes. While the next most abundant phyla is dependent on diet type – Bacteroidetes is the second most abundant phyla for mice adhering to a normal diet, while this phyla was significantly diminished in GFD mice. **(D)** There is no significant variation between *NLRP12^-/-^* mice and wild-type mice in the observed species present, either for normal diet or GFD. **(E)** There is a clear clustering of the groups in PCoA analysis with clusters occurring based on diet-type. GFD, *n* = 7*;* normal diet, *n* = 7. *p < 0.05, **p < 0.01, ***p < 0.001, ****p < 0.0001.

NLRP12 functions as a negative regulator of noncanonical NF-κB signaling, making *Nlrp12^-/-^* mice more susceptible to gastrointestinal inflammation in the context of inflammatory bowel disease, colitis-associated cancer, and obesity as shown in previous studies (28, 34, 38, 49, 59). Compared to NLRX1-deficient mice, the absence of NLRP12 had minimal effects on the gut microflora. There was no significant variation in *Nlrp12^-/-^* and *Nlrp12^+/+^* littermate control mice fed a normal diet or following GFD in observed species, Shannon diversity, or relative abundance of any major phyla (**Figures 6A, B, D**). This suggests that genetic NLRP12-deficiency does not inherently alter the gut microbiome composition. Similar trends were observed in both comparing *Nlrp12^-/-^* normal diet to *Nlrp12^-/-^* GFD and comparing *Nlrp12^+/+^* normal diet to *Nlrp12^+/+^* GFD, in which number of observed species and Shannon diversity were significantly decreased in GFD mice regardless of genetic deficiency (**Figures 6A, B**). Likewise, there was no significant variation between *Nlrp12^-/-^* and *Nlrp12^+/+^* fed a GFD (**Figures 6A, B, D**). Upon further investigation of the microbial composition, the relative abundance of bacteria belonging to the Actinobacteria phyla was increased in GFD mice, while Bacteroidetes was decreased regardless of genetic status (**Figures 6C, D**). The relative abundance of Firmicutes and Proteobacteria were also not found to be significant for any of the comparisons (**Figures 6C, D**).

**Figure 7:**
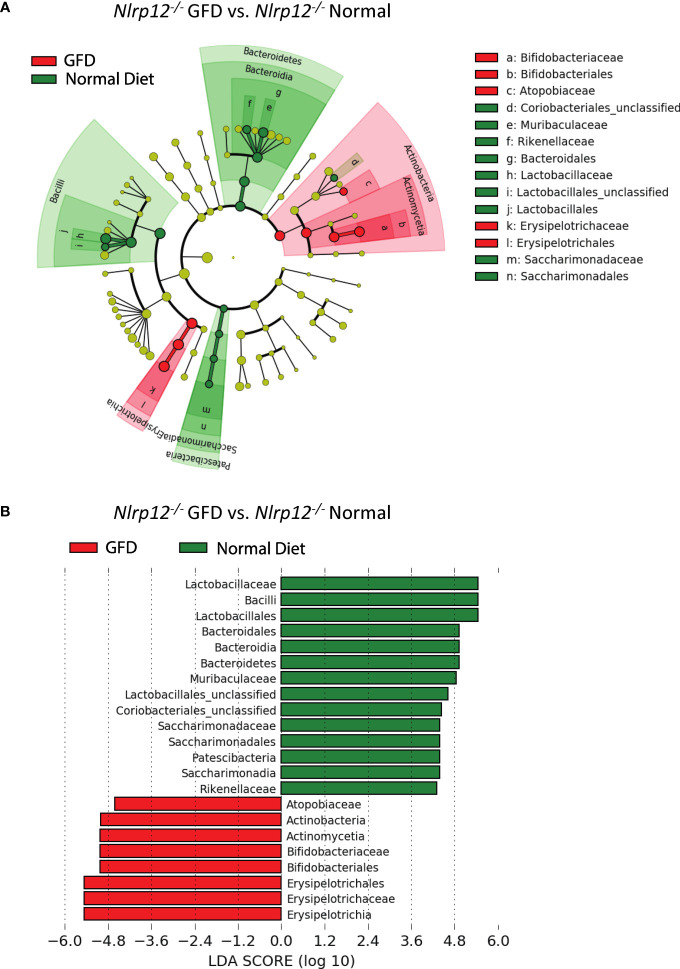
The Effects of GFD on the Microbiome of NLRP12-deficient Mice. **(A)** Cladogram displaying differential bacterial abundance in *NLRP12^-/-^* mice fed GFD (red) and normal diet (green). **(B)** LEfSe differential analysis displaying bacterial abundance in *NLRP12^-/-^* mice fed GFD (red) and normal diet (green) reveals that significant differences exist between the microbial populations. The graph was generated using the LEfSe program. GFD (red), *n* = 7*;* normal diet (green), *n* = 7.

Further evaluation of the distinctiveness of these microbiome compositions show two overlapping clusters in PCoA is driven by dietary intervention (**Figure 6E**; **Supplemental Table 3**). These community structures strongly clustered based on diet status. Following dietary intervention in *Nlrp12^-/-^* mice, LefSE analyses indicate that these two microbiomes indeed have unique identifiers (**Figure 7**). Mice fed a GFD were characterized by Bifidobacteriales and Bifidobacteriaceae (**Figure 7**), with some strains commonly administered as probiotics. Mice fed a normal diet have increased colonization of Lactobacillaceae, Lactobacillales, unclassified Lactobacillales, and Bacilli, which generally include symbiotic bacteria commonly used for probiotics (**Figure 7**). This trend mirrors what we have observed in wild-type mice (**Figure 3**) in that wild-type mice administered long-term GFD has apparent loss of beneficial gut microbes. It is important to indicate that the microbiomes of *Nlrp12^-/-^* mice fed a GFD were also represented by bacteria belonging to Bifidobacteriales and Bifidobacteriaceae, which also include probiotic bacteria. Our findings demonstrate that NLRP12-deficiency does not inherently alter the gut microbiome (**Figure 7**). Rather, the microbiome of NLRP12-deficient mice resembles that of wild-type mice and any alterations observed in the bacterial composition is driven by diet.

### GFD Alters Metabolite Production in Mice Fed GFD

**Figure 8:**
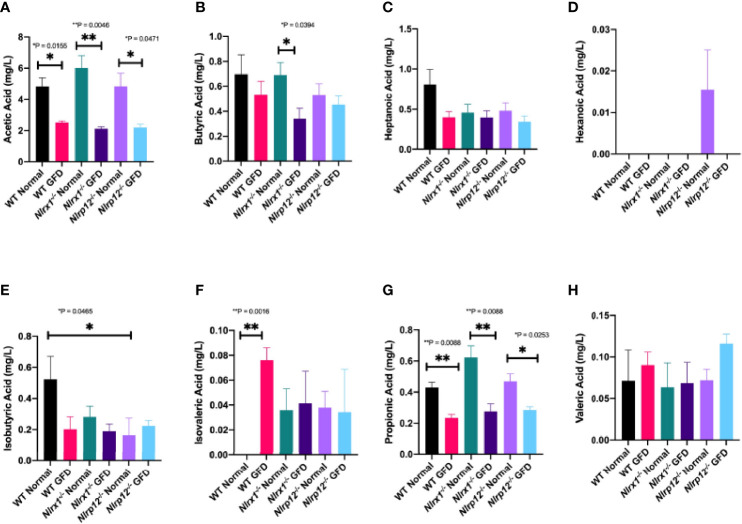
SCFA and MCFA Levels Significantly Vary Based on Diet. Abundance of the **(A)** acetic acid, **(B)** butyric acid, **(C)** heptanoic acid, **(D)** hexanoic acid, **(E)** isobutyric acid, **(F)** isovaleric acid, **(G)** propionic acid, and **(H)** valeric acid in mg/L of feces in wild-type, *Nlrx1^-/-^, and Nlrp12^-/-^* mice*. n* = 3-7. *p < 0.05, **p < 0.005.

We found that short-chain fatty acid (SCFA) and medium-chain fatty acid (MCFA) levels significantly vary based on diet type (**Figure 8**). Diet associated alterations in metabolite production were most prominent for acetic and propionic acid (**Figures 8A, G**). Regardless of genotype, exposure to a GFD significantly decreases the production of acetate and propionate (**Figures 8A, G**). Acetate levels were significantly decreased between wild-type (*p* = 0.0155), *Nlrx1^-/-^* (*p* = 0.0046), and *Nlrp12^-/-^* (*p* = 0.0471) mice on a normal diet compared to respective strains on GFD (**Figure 8A**). Propionate levels were significantly decreased between wild-type (*p* = 0.0088), *Nlrx1^-/-^* (*p* = 0.0088), and *Nlrp12^-/-^* (*p* = 0.0253) mice on a normal diet compared to GFD (**Figure 8G**). We observed decreased levels of butyrate in *Nlrx1^-/-^* GFD compared to *Nlrx1^-/-^* normal (**Figure 8B**). Minimal deviations in isobutyric, isovaleric, and hexanonic acids are observed, which may not necessarily be genotype independent (**Figures 8D-F**). These aberrant fluctuations in SCFA/MCFA levels provides further insight to the shifts in the microbiota following GFD.

## Discussion

A delicate balance of innate immune signaling mechanisms is needed to maintain gut health and any perturbations, particularly in NLR signaling as demonstrated here, have the potential to disturb the symbiotic relationship between host and microbiota by promoting hyperinflammatory signaling. Only a few studies have evaluated NLRs in association to CeD. The best described NLR thus far is NOD1, which has been shown to be downregulated in CeD patients (67); whereas, NOD2 and CIITA expression has been reported to be unchanged (68). Studies have yet to evaluate the gut microbiome following NLRX1-deficiency and CeD pathogenesis; however, this negative regulatory NLR, NLRX1, has been evaluated in other gastrointestinal disorders. One study found *Nlrx1^-/-^* mice to have unique microbiomes that are characterized by colitogenic microbes, particularly belonging to the bacterial taxa *Veillonella* and Clostridaiales (37). These microbiomes characteristic of NLRX1-deficiency are in part due to impaired intestinal epithelial cell function and metabolite production, therefore increasing gut barrier permeability and leading to greater colonization of opportunistic pathogens (37). Our data also demonstrate that *Nlrx1^-/-^* mice have an inherently unique gut microbiome that is further shifted following a GFD (**Figures 4**, **5**; **Supplemental Figure 2**; **Supplemental Table 2**). With this distinctive shift in the microbial community, we observed that *Nlrx1^-/-^* mice had a slight increase in alpha diversity compared to mice with intact NLR signaling (**Figures 4A, B**). Although enhanced bacterial diversity would suggest enhanced ecological fitness, further examination of the bacterial composition of these genetically impaired mice shows increased relative abundance of opportunistic bacteria that are associated with intestinal inflammation, disorders, and infections. Such opportunistic bacteria include unclassified Enterococcaceae (**Figure 5C**). Enterococcaceae are often found in the gut as commensals yet are opportunistic and elicit infections upon penetration of the gut epithelial barrier (69, 70). Given the NLR-deficiency of these mice, deletion of the negative regulatory NLRX1 is anticipated to further amplify pro-inflammatory signaling in the gastrointestinal tract. Our 16S rRNA sequencing data characterizing the microbiome suggests that loss of NLRX1 alters the gut microbiota in response to administration of a GFD. We further speculate that loss of NLRX1 is conducive towards hyperinflammation as this negative regulator of NF-κB signaling is rendered inactive allowing for overzealous inflammatory signaling, which further alters the microbiome and likely exacerbates GFD-mediated dysbiosis.

Unlike NLRX1, we did not observe significant changes in the NLRP12-deficient microbiome. The microbiome of NLRP12-deficient mice has been previously characterized as having reduced bacterial diversity and decreased abundance of the protective commensal Lachnospiraceae contributing to colonic inflammation, as in human Ulcerative Colitis patients (34, 71). The microbiota of NLRP12-deficient mice spontaneously promotes excessive weight gain as a model for obesity and also elicits pro-inflammatory signaling in the colon (38). Further, these mice had diminished bacterial diversity, which is commonly associated with dysbiosis and intestinal inflammation (38). These phenotypes were resolved in mice either treated with antibiotics or housed in germ-free conditions (38). Our data, however, showed little to no variation in *Nlrp12^-/-^* normal compared to *Nlrp12^+/+^* mice or *Nlrp12^-/-^* GFD in comparison to *Nlrp12^+/+^* GFD at a normal baseline or following GFD (**Figures 6**, **7**; **Supplemental Table 3**). Our findings indicate that NLRP12-deficiency has minimal effects on regulating the gut microbiota in healthy mice or in GFD-fed mice. Differences in our findings and previous literature may be attributed to our work being conducted at a different facility with different vivarium conditions, use of littermate control animals, or differences in experimental design, including but not limited to primer choice, sequencing methods, and computational analysis. Murine microbiome studies are carefully designed to take into consideration and control for several different factors such as littermate controls, genetic background, maternal inheritance, and vivarium differences in order to ensure reproducibility and draw conclusions that accurately reflect the true nature of these biological processes, all of which were controlled for in our study to limit unaccounted for variation under our specific experimental conditions.

Beyond evaluating the role of NLR signaling in regulating the microbiome, we evaluated the potential effects of GFD in a healthy population that is void of gluten-sensitivities. Of interest, there is an ever-increasing popularity in “elimination diets”. Two of these most popular trends include GFD and cereal-free Paleo diets that promote the exclusion of gluten or cereal grains, respectively, which claim to minimize inflammation and the onset of inflammatory diseases in otherwise healthy individuals. However, claims of such long-term health benefits have yet to be properly and convincingly studied in people overtime. Instead, unnecessary adherence to GFD restricts food sources rich in prebiotics that are necessary for proper bacterial fermentation and metabolic activity. Additionally, healthy individuals, who have neither gluten-sensitivities nor CeD, are sacrificing well-balanced diets for strict adherence to dietary regimens that increase their risk of malnutrition and mineral/vitamin deficiencies. As a potential model for this exclusion diet in healthy individuals, wild-type mice fed GFD encompassed a significant shift in their gut microflora (**Figure 3**). The gut flora of wild-type mice was not robust enough to withstand the effects of a GFD diet, as depicted by distinctive microbiome composition and metabolite levels (**Figures 3**, **8**). This rapid shift in the gut microbiome following GFD may serve as precedent to dysbiosis. In wild-type mice fed GFD for instance there was increased Eryipelotrichaceae family *Faecalibaculum* genus observed. Of interest, bacteria belonging to *Faecalibaculum* genus and Erysipelotrichaceae family have ties to IBD and colitogenic properties (34, 63, 72) ever-highlighting the potential association between IBD and CeD enteropathies, especially given the NLR-deficient mouse models. Our data highlights emerging concerns that may be associated with strict adherence to exclusion diets for individuals void of gluten sensitivities, where loss of beneficial gut microbes and bacterial diversity primes the gut microenvironment for pro-inflammatory signaling attributed to several gastrointestinal pathologies.

Although adherence to a gluten-free diet is the most effective treatment regimen for CeD patients, it is not without faults. Infants carrying the HLA-DQ2 haplotype are genetically predisposed to CeD with their developing gut microbiomes being associated with increased proportions of bacteria belonging to the *Clostridium* species (Firmicutes) and Enterobacteriaceae family (Proteobacteria), and decreased proportions of beneficial *Bifidobacterium* species (Actinobacteria) than infants considered low risk (73). Notably, *Bifidobacterium* is commonly administered as a probiotic to decrease levels of pro-inflammatory TNF, IFN-gamma, and IL-2 (74). We found the Bifidobacterium family (Actinobacteria) to be increased in GFD fed NLRP12-deficient mice and mice with intact NLR signaling. However, of interest, NLRX1-deficient mice had the highest levels of *Bifidobacterium* at homeostatic conditions when fed a normal diet but was greatly reduced by GFD (**Figure 7**). Co-administration of *Bifidobacterium* as a probiotic in CeD pediatric patients currently adhering to a GFD between 6 months to 15 years was found to restore the gut microbiota and decrease the previously heightened levels of acetate and propionate SCFAs (74). In order to maximize the effectiveness of GFD in alleviating gastrointestinal issues, co-administration of GFD with probiotics should be considered to potentially restore the loss of commensal and beneficial microbes. Alternatively, food technology is currently developing “pseudocereal” formulations of amaranth (75), quinoa (75), buckwheat (75), spirulina (76) and red rice flour (77) that can replace gluten and exhibit prebiotic effects on probiotic bacteria.

Metabolite production is another field of interest where it is predicted that a SCFA fingerprint exists as a biomarker for such gastrointestinal diseases; however, a single SCFA fingerprint has yet to be characterized in CeD patients. Previous literature has reported increased levels of acetic acid (acetate), propionic acid (propionate), and total SCFAs in CeD patients (74, 78–81). However, it is unclear if these changes are associated with CeD or the GFD adopted by these patients. Consistently, we observed decreased acetate and propionate levels following GFD regardless of genetic background (**Figure 8**). Healthy adults on a GFD had decreased levels of SCFAs compared to those on a normal diet (78), which is consistent with our observations comparing GFD and normal diet wild-type mice (**Figure 8**). Comparison of SCFAs in CeD pediatric patients treated with GFD for at least one year, GFD less than one year, and healthy children found that CeD patients that adhered to a GFD for greater than a year closely resembled SCFA levels of healthy children, while CeD patients treated for less than one year had significantly increased SCFA levels (79). We observed a similar trend in our murine study, where GFD mice regardless of NLRX1 or NLRP12 genotype had decreased metabolite levels (**Figure 8**). Acetate levels were significantly decreased between each genotype (wild-type, *Nlrx1^-/-^*, and *Nlrp12^-/-^*) on a GFD compared to normal diet (**Figure 8A**). Acetate levels are the most abundant SCFA at >50% and are critically involved in the ability of Bifidobacterium to inhibit enteropathogens, therefore ensuring symbiosis of the gut microbiome (78). In Nlrx1^-/-^ mice, we observed decreased levels of butyrate in mice fed GFD compared to normal diet (**Figure 8B**). Steady levels of butyrate are beneficial in maintaining the gut mucosal barrier and reducing the risk of several gastrointestinal disorders (82–84). Along with butyrate, propionate is critical for the production of gut hormones that regulate appetite and is also involved in the activation of kinase-mediated pathways (85, 86). The complete omission of gluten results in a collateral loss of prebiotics and a deficit of micronutrients that are utilized in the metabolic activity of microorganisms. Due to this loss, the probiotic effect of beneficial microbes is significantly impaired resulting in expansion of pathogenic/opportunistic microbes.

Overall, the culmination of our findings suggests that GFD as a dietary intervention, although the standard of care for CeD and NCGS patients, has limitations. This treatment regimen completely omits food sources containing gluten to prevent any robust autoimmune responses to partially digested gluten fragments and gliadins. While this is a critical avoidance therapy necessary to control these diseases, the significant change from a normal diet disrupts the delicate homeostasis of the microbiome and shifts it towards dysbiosis. Due to this shift in food intake, diet-associated changes to the microflora also interfere with metabolite production. Specifically, GFD decreases SCFA production, which is commonly upregulated in CeD patients, but at the cost of microbiome symbiosis. Our study reveals that adherence to a GFD in otherwise healthy mice results in the loss of beneficial microbes, which suggests that administration of GFD could be paired with probiotics to further promote gut health by reintroducing loss populations of commensals. Using murine models to further explore the role of NLRX1 and NLRP12 deficiencies in microbiota maintenance, we found the gut microbiome of NLRP12-deficient mice did not significantly differ from wild-type mice with intact NLR signaling. However, we found that NLRX1-deficient mice have a genetically inherent gut microbiome that may be primed for dysbiosis following GFD. This, therefore, unveils a potential role of appropriate NLRX1 signaling in regulating the gut microbiome and its potential contribution to CeD and NCGS pathogenesis.

## Data Availability Statement

The datasets presented in this study can be found in online repositories. The names of the repository/repositories and accession number(s) can be found below: https://www.ncbi.nlm.nih.gov/, PRJNA808753.

## Ethics Statement

The animal study was reviewed and approved by Virginia Tech Institutional Animal Care and Use Committee (IACUC).

## Author Contributions

Design and execution of the experiments were conducted by HM, KE, YL, and MN-S. KE was responsible for mouse breeding, genotyping, and general husbandry. Experimental data interpretation was conducted by HM, YL, PW, and IA. HM, KE, and YL contributed equally to this work and share first authorship. PW and IA contributed equally to this work and share senior authorship. All authors contributed to the writing and revising of the manuscript. All authors contributed to the article and approved the submitted version.

## Funding

Grants were awarded from the US National Institutes of Health (IA; R03 DK105975 and K01 DK092355); the *Via* College of Osteopathic Medicine (VCOM) One Health Center Seed Funding (IA); Virginia Maryland College of Veterinary Medicine Internal Research Competition (IA), and the Virginia Tech Institute for Critical Technology and Applied Sciences (IA). This work was supported, in part, by the Intramural Research Program of the National Institute of Environmental Health Sciences (ES101965 to PW).

## Conflict of Interest

The authors declare that the research was conducted in the absence of any commercial or financial relationships that could be construed as a potential conflict of interest.

## Publisher’s Note

All claims expressed in this article are solely those of the authors and do not necessarily represent those of their affiliated organizations, or those of the publisher, the editors and the reviewers. Any product that may be evaluated in this article, or claim that may be made by its manufacturer, is not guaranteed or endorsed by the publisher.

## Supplementary Material

Supplementary Figure 1 Potential Downstream Targets Implicated by Impaired NLR Signaling in CeD. The subset of genes evaluated here are commonly expressed upon pro-inflammatory signaling downstream of NLR signaling. The majority of genes were upregulated with the exception of *UEV1A*, *RELB, IL18*, and *CXCL12* in active CeD patients. * *p* < 0.05.

Supplementary Figure 2 Diet Alters the Gut Microbiome of NLRX1-deficient Mice at the Genus Level. Relative abundance of two genera found at significantly increased levels in *Nlrx1^-/-^* mice fed GFD. n = 7 mice per group. **** p < 0.0001*.
