# Supplementary figures and images for: NLRX1 Deficiency Alters the Gut Microbiome and Is Further Exacerbated by Adherence to a Gluten-Free Diet
Source: Front Immunol. 2022 Apr 28;13:882521. doi: 10.3389/fimmu.2022.882521 (PMC9097893; doi:10.3389/fimmu.2022.882521)

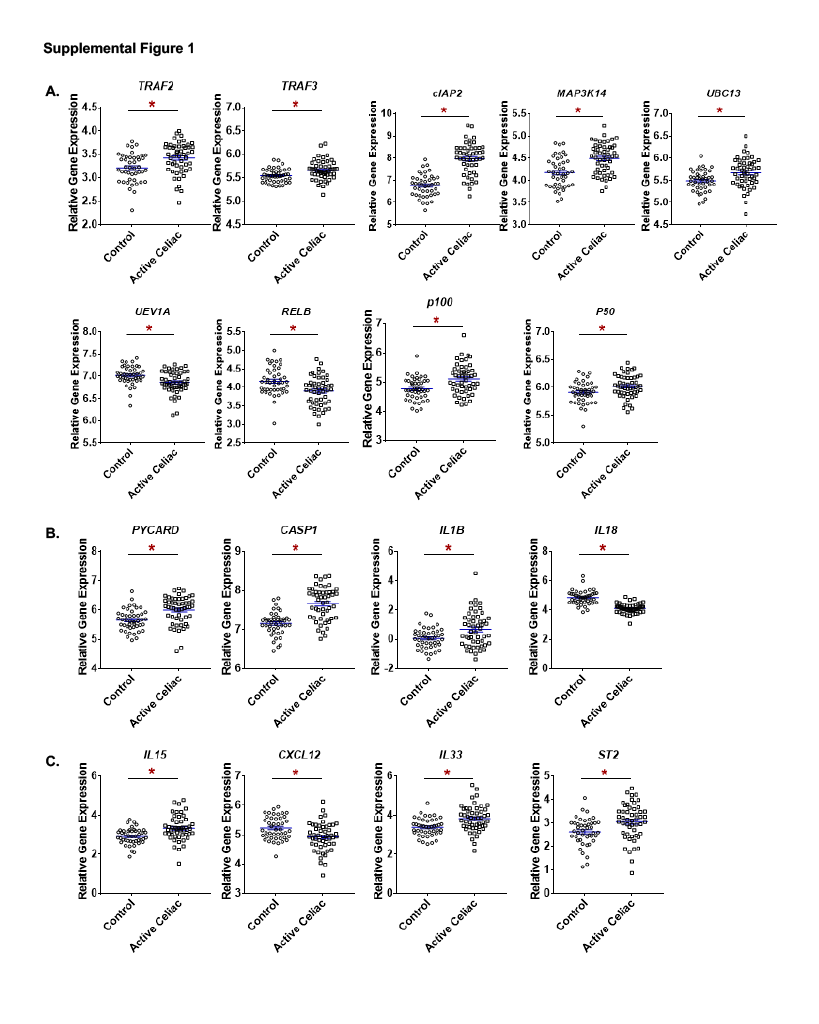

Supplement: Supplementary Figure 1 — Potential Downstream Targets Implicated by Impaired NLR Signaling in CeD. The subset of genes evaluated here are commonly expressed upon pro-inflammatory signaling downstream of NLR signaling. The majority of genes were upregulated with the exception of UEV1A, RELB, IL18, and CXCL12 in active CeD patients. * p < 0.05. [file Image_1.tiff]

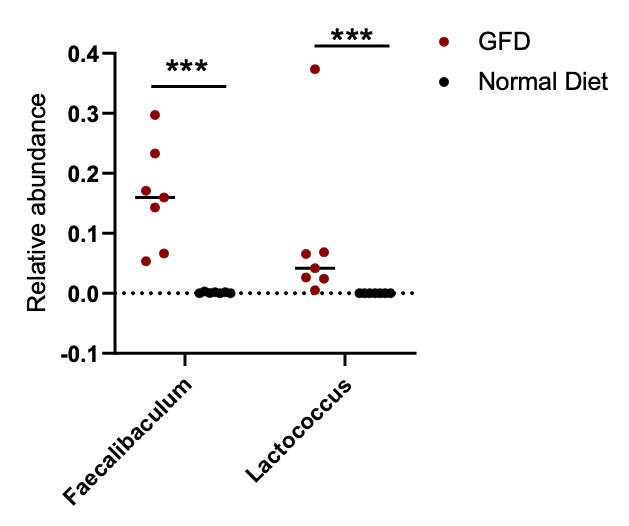

Supplement: Supplementary Figure 2 — Diet Alters the Gut Microbiome of NLRX1-deficient Mice at the Genus Level. Relative abundance of two genera found at significantly increased levels in Nlrx1-/- mice fed GFD. n = 7 mice per group. *** p < 0.0001. [file Image_2.tiff]
