# Supplementary material for: NLRX1 Deficiency Alters the Gut Microbiome and Is Further Exacerbated by Adherence to a Gluten-Free Diet
Source: Front Immunol. 2022 Apr 28;13:882521. doi: 10.3389/fimmu.2022.882521 (PMC9097893; doi:10.3389/fimmu.2022.882521)
Supplement: Supplementary file 3 [file Table_1.docx]

**Supplemental Table 1.** Ingredient List for GFD and Normal Diet

| NORMAL DIET | | GLUTEN-FREE DIET | |
| --- | --- | --- | --- |
| *Tekland Global 18% Protein Rodent Diet* | | *Open Source Diets AIN-76A* | |
| Ingredient | **g/kg** | **Ingredient** | **g/kg** |
| *Ground wheat* | Proprietary information that cannot be disclosed by vendor | *Casein, Lactic, 30 mesh* | 200.00 |
| *Ground corn* |  | *Methionine, DL* | 3.00 |
| *Wheat middlings* |  | *Sucrose, Fine granulated* | 500.00 |
| *Dehulled soybean meal* |  | *Starch, Corn* | 150.00 |
| *Corn gluten meal* |  | *Solka Floc, FCC200* | 50.00 |
| *Soybean oil* |  | *Corn oil* | 50.00 |
| *Calcium carbonate* |  | [*S10001*](https://researchdiets.com/en/formulas/S10001) *Mineral Mix* | 35.00 |
| *Dicalcium phosphate* |  | [*V10001*](https://researchdiets.com/en/formulas/V10001) *Mineral Mix* | 10.00 |
| *Brewers dried yeast* |  | *Choline bitartrate* | 2.00 |
| *Iodized salt* |  |  |  |
| *L-lysine* |  |  |  |
| *DL-methionine* |  |  |  |
| *Choline chloride* |  |  |  |
| *Kaolin* |  |  |  |
| *Magnesium oxide* |  |  |  |
| *Vitamin E acetate* |  |  |  |
| *Menadione sodium bisulfite complex* |  |  |  |
| *Manganous oxide* |  |  |  |
| *Ferrous Sulfate* |  |  |  |
| *Zinc oxide* |  |  |  |
| *Niacin* |  |  |  |
| *Calcium pantothenate* |  |  |  |
| *Vitamin A acetate* |  |  |  |
| *Calcium iodate* |  |  |  |
| *Vitamin B12 supplement* |  |  |  |
| *Folic acid* |  |  |  |
| *Biotin* |  |  |  |
| *Vitamin D3 supplement* |  |  |  |
| *Cobalt carbonate* |  |  |  |
| Macronutrients | **Kcal%** | **Macronutrients** | **Kcal%** |
| Carbohydrate | 58 | **Carbohydrate** | 67.7 |
| Protein | 24 | **Protein** | 20.8 |
| Fat | 18 | **Fat** | 11.5 |
