# Supplementary material for: NLRX1 Deficiency Alters the Gut Microbiome and Is Further Exacerbated by Adherence to a Gluten-Free Diet
Source: Front Immunol. 2022 Apr 28;13:882521. doi: 10.3389/fimmu.2022.882521 (PMC9097893; doi:10.3389/fimmu.2022.882521)
Supplement: Supplementary file 4 [file Table_2.docx]

**Supplemental Table 2.** AMOVA Analysis for NLRX1 PcoA Plot

| *Nlrx1* Comparisons | Fs | p-value |
| --- | --- | --- |
| Nlrx1+/+ Normal vs Nlrx1 +/+ GFD | 17.7 | 0.001 |
| Nlrx1+/+ Normal vs Nlrx1 -/- Normal | 3.39 | 0.004 |
| Nlrx1+/+ Normal vs Nlrx1 -/- GFD | 10.9 | <0.001 |
| Nlrx1 -/- Normal vs Nlrx1 -/- GFD | 15.5 | 0.002 |
| Nlrx1 -/- Normal vs Nlrx1 +/+ GFD | 28.1 | <0.001 |
| Nlrx1 +/+ GFD vs Nlrx1 -/- GFD | 9.9 | 0.001 |
