# Supplementary material for: NLRX1 Deficiency Alters the Gut Microbiome and Is Further Exacerbated by Adherence to a Gluten-Free Diet
Source: Front Immunol. 2022 Apr 28;13:882521. doi: 10.3389/fimmu.2022.882521 (PMC9097893; doi:10.3389/fimmu.2022.882521)
Supplement: Supplementary file 5 [file Table_3.docx]

**Supplemental Table 3.** AMOVA Analysis for NLRP12 PcoA Plot

| *Nlrp12* Comparisons | Fs | p-value |
| --- | --- | --- |
| Nlrp12+/+ Normal vs Nlrp12 +/+ GFD | 16.5 | 0.002 |
| Nlp12+/+ Normal vs Nlrp12 -/- Normal | 0.9 | 0.492 |
| Nlrp12+/+ Normal vs Nlp12 -/- GFD | 16.7 | <0.001 |
| Nlrp12 -/- Normal vs Nlp12 -/- GFD | 18.6 | 0.001 |
| Nlp12 -/- Normal vs Nlp12 +/+ GFD | 18.5 | 0.001 |
| Nlp12 +/+ GFD vs Nlrp12 -/- GFD | 3.9 | 0.004 |
